# Anaesthetic management of an unusual complication of myringoplasty

**DOI:** 10.4103/0019-5049.63651

**Published:** 2010

**Authors:** Sandhya Agarwal, Ritu Aggarwal, Savita Babbar

**Affiliations:** Departments of Anaesthesiology and Critical Care, Deen Dayal Upadhyay Hospital, Hari Nagar, New Delhi, India

**Keywords:** Craniotomy, intraparenchymal haematoma, myringoplasty, neuroanaesthesia

## Abstract

A young male patient was undergoing myringoplasty for right ear chronic suppurative otitis media. While drilling in the middle ear cavity, duramater was breached accidentally. Surgeons were, however, allowed to complete the procedure. Keeping the seriousness of such a complication in mind, an urgent neurosurgical intervention was sought and noncontrast computed tomography head scan was done to analyse the extent of the injury. Osteoplastic craniotomy had to be performed subsequently to evacuate the contusional haematoma over the right temporoparietal region. Throughout the procedure, patient's vitals were monitored vigilantly to prevent any further deterioration of his condition. All the available resources were tapped judiciously to maintain intracranial pressure within normal limits. With a quick responsiveness on the part of the anaesthesia team, an active decision making, appropriate and remarkable anaesthetic management both intra and postoperatively, and good ICU care, a young patient could be salvaged and discharged successfully within a week with no immediate or residual complications related to myringoplasty or any neurological deficit.

## INTRODUCTION

Chronic suppurative otitis media (CSOM) has been an important cause of middle ear disease since the prehistoric times. The complication rate of myringoplasty is low in all series. Kotecha *et al*. reported one facial nerve palsy in 1070 patients and one extradural abscess.[1] However, duramater rupture with contusional haematoma formation over temporo-parietal region during a myringoplasty procedure has previously not been described. In our case, surgeons reported a sudden breach of duramater over the aditus during the index procedure. With a good neuroanaesthetic management, patient was managed for the above complication and successfully extubated.

## CASE REPORT

A 16-year-old, 70-kg ASA grade I male was undergoing myringoplasty for right ear CSOM in routine elective operation theatre under general anaesthesia. Unexpectedly, the duramater was breached over the aditus after 2 hours of initiating the surgery, which was recognised as a sudden give way by the otolaryngiologists. Keeping the impending cerebral compromise in mind, we sought an emergency neurosurgical consultation and decided for an urgent noncontrast computed tomography (NCCT) head scan. Meanwhile, temporalis muscle flap was placed over the breached dura by the operating surgeon. The wound was closed in two layers over gel foam and a drain. The patient was electively ventilated in ICU while awaiting NCCT head scan report with Assist Control Ventilation (volume cycled) and was maintained on continuous intravenous infusions of inj. Midazolam 0.25 mcg/kg/minute and inj. Vecuronium 1 mcg/kg/minute. Vitals were maintained within normal limits. The patient was catheterised, a nasogastric tube inserted and fundoscopy done to rule out papilloedema.

**Figure 1:**
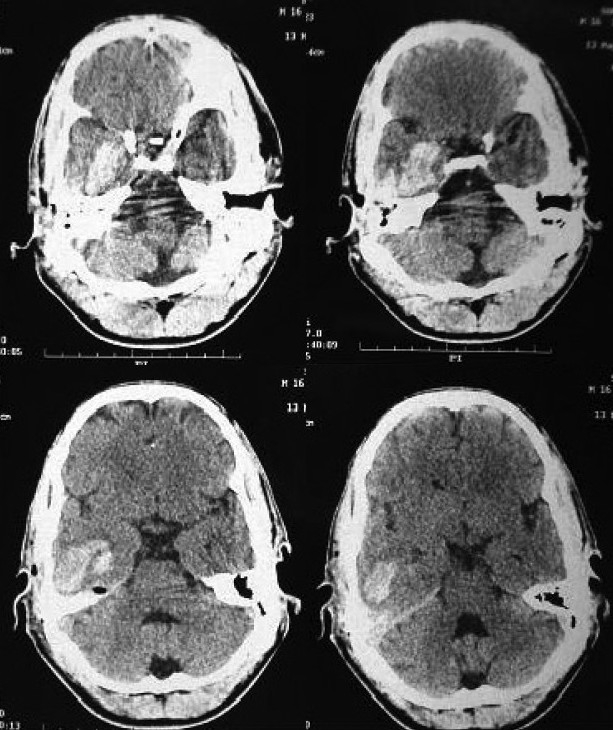
NCCT head scan showing intraparenchymal haematoma of size 4.5 x 2 x 3 cm

NCCT head scan revealed [Figure 1]

- intraparenchymal haematoma of size 4.5 × 2 × 3 cm with minimal mass effect and peri-lesional oedema with few foci of pneumocephalus in right temporal region,
- extra-axial bleed (subdural) in right tentorium cerebelli and
- bony defect in right petrous temporal bone with fluid in middle and external ear cavity.

In ICU, complete haemogram, prothombin time (PT), activated partial thromboplastin time (aPTT) and international normalised ratio (INR) were done. Patient was given inj. Phenytoin 800 mg i.v. (loading dose) and inj. Vit. K one amp. i.m. along with the antibiotic cover.

**Figure 2:**
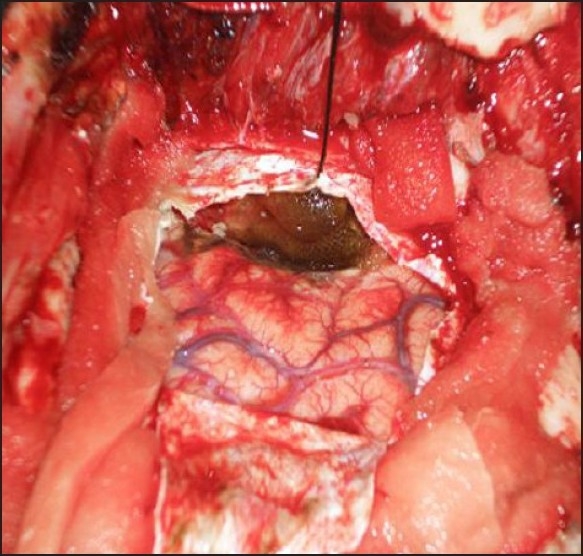
Intracranial haematoma following dura breach (as seen during craniotomy)

An urgent right temporo-parietal osteoplastic craniotomy was planned for the drainage of contusional haematoma in 15° head up position [Figure 2]. High risk consent was taken. Central line was inserted through right subclavian vein. Inj. Fentanyl 2 mcg/kg i.v. bolus followed by 0.5 mcg/kg hourly was given; inj. Midazolam and vecuronium infusions were continued. The patient was ventilated with 100% oxygen and isoflurane 0.4–0.6 vol.% maintaining normocapnia throughout. Inj. Mannitol (20%) 1 g/kg i.v. was given over 15–20 minutes after skin incision. Surgery lasted for 3 hours. Intraoperative blood loss was 800 ml. Intravenous fluids consisted of 1500 Normal Saline, 500 ml Dextrose Normal Saline and 2 units whole blood (Hb was 10.5 g/dl) to maintain haematocrit above 30%. Central venous pressure (CVP) was maintained at 8–10 cm H_2_O, urine output was adequate. The above regimen was implemented to maintain the vitals and cerebral haemodynamics within physiological limits. Inj. Ondansetron 6.0 mg i.v. (for postoperative nausea and vomiting) and inj. Diclofenac sodium 75 mg i.m. (for postoperative analgesia) were administered. Inj. Vecuronium and inj. Midazolam infusions were stopped. When patient was fully conscious with good cognition, adequate protective airway reflexes and motor power, he was extubated after giving inj. Glycopyrrolate 5 mcg/kg i.v. and inj. Neostigmine 50 mcg/kg i.v. There were no signs of any residual neurological deficit. The patient was observed in ICU. Subsequent NCCT head scan was normal. Treatment regimen consisted of inj. Amikacin, inj. Augmentin, inj. Phenytoin, inj. Mannitol and syp. Citicoline. The patient was discharged on the seventh postoperative day.

## DISCUSSION

Variations in the anatomy of the temporal bone, such as anteriorly placed lateral venous sinus, low middle cranial fossa dura or a prominent Korner's septum create additional difficulties for the inexperienced surgeon, leading to various complications.[2] The effects of intracranial haematomas depend, particularly, on the speed with which they arise. Fast occurring intracerebral haematoma (as in our case) can lead to massive neurological impairment and potentially acutely life threatening increase in intracranial pressure (ICP) necessitating urgent surgical decompression. Secondary insults to the already injured brain include: intracranial – increased ICP, epilepsy, vasospasm, herniation of falx, tentorium or foramen magnum, craniotomy, midline shift due to tearing of cerebral vessels; extracranial – hypercapnia/hypoxaemia, hypotension/hypertension, hypoglycaemia/hyperglycaemia, low cardiac output and hypo-osmolality.[3]

Major goals during anaesthesia include (1) maintenance of adequate perfusion and oxygenation of normal brain, (2) optimising operative conditions to facilitate resection, (3) ensuring rapid emergence of anaesthesia at the conclusion of the procedure to facilitate neurological assessment, and when appropriate, (4) accommodating intraoperative electrophysiological monitoring.[4] Since CVP, mean arterial pressure (MAP) and fundoscopy were normal with no signs of Cushing's triad, we assumed that the ICP was within normal limits. Bispectral index (BIS) was used to monitor the depth of anaesthesia. Internal jugular vein cannulation was avoided as it could interfere with the cerebral venous drainage. Instead, right subclavian vein was used to measure CVP, for venous access and treat venous air embolism (VAE) if it occurred intraoperatively. Apart from increasing cerebral metabolic rate of oxygen (CMRO_2_), cerebral blood flow (CBF) and ICP, since N_2_O is 35 times more diffusible than nitrogen, pneumocephalus and VAE (if it occurs) can expand dangerously and, therefore, was avoided. Oxygen–air mixture is preferable but we used 100% oxygen along with isoflurane 0.4–0.6 vol.% as air was not available at our institution. Isoflurane produces a reduction in the CMRO_2_ which is greater than that produced by equivalent Minimum Alveolar Concentration (MAC) values of halothane, but the CBF increase with isoflurane is minimal below 1.1 MAC.[5] The net vasodilating effect of equi-MAC concentrations of isoflurane, desflurane and sevoflurane is less in humans than that of halothane, and the former are therefore preferable if a volatile anaesthetic is to be used in the setting of impaired intracranial compliance.[6]

Veselis and colleagues, using PET (Positron Emission Tomography) observed a global reduction in CBF of 12% after 0.15 mg/kg of inj. Midazolam to awake healthy human volunteers and noted that the decrease occurred preferentially in brain regions associated with arousal, attention and memory.[7] Further, CO_2_ responsiveness is preserved.[8] Inj. Fentanyl also causes a moderate decrease in CBF and CMRO_2_ and inj. Vecuronium, as such, has no cerebral and few systemic side effects.

Though we used the above technique, target controlled infusion (TCI) or total intravenous anaesthesia (TIVA) using inj. Propofol are being used at some centres, more so during awake craniotomies. At the end of surgery, a rapid recovery of consciousness is essential so that the level of response can be assessed along with Glasgow Coma Scale and to rule out any neurological deficits. Use of a good antibiotic regimen, phenytoin (antiepileptic), mannitol (osmolar diuretic), dihyropyridine calcium channel blocker like nimodipine (prevents cerebral vasospasm) and a vitamin B choline like citicoline (neuroprotective agent), helps in rapid postoperative recovery.

Thus, a seemingly normal procedure can pose a challenge to an anaesthesiologist's competence. When such complications occur, he should be able to take prompt decisions, act swiftly and use all the available resources optimally to affect a good patient outcome.
